# *(n* − 1)-Step Derivations on *n*-Groupoids: The Case *n* = 3

**DOI:** 10.1155/2014/726470

**Published:** 2014-07-08

**Authors:** N. O. Alshehri, Hee Sik Kim, J. Neggers

**Affiliations:** ^1^Department of Mathematics, King Abdulaziz University, Faculty of Science for Girls, Jeddah, Saudi Arabia; ^2^Department of Mathematics, Hanyang University, Seoul 133-791, Republic of Korea; ^3^Department of Mathematics, University of Alabama, Tuscaloosa, AL 35487-0350, USA

## Abstract

We define a ranked trigroupoid as a natural followup on the idea of a ranked bigroupoid. We consider the idea of a derivation on such a trigroupoid as representing a two-step process on a pair of ranked bigroupoids where the mapping *d* is a self-derivation at each step. Following up on this idea we obtain several results and conclusions of interest. We also discuss the notion of a couplet (*D*, *d*) on *X*, consisting of a two-step derivation *d* and its square *D* = *d* ∘ *d*, for example, whose defining property leads to further observations on the underlying ranked trigroupoids also.

## 1. Introduction

The notion of derivations arising in analytic theory is extremely helpful in exploring the structures and properties of algebraic systems. Several authors [1, 2] studied derivations in rings and near rings. Jun and Xin [3] applied the notion of derivation in ring and near ring theory to BCI-algebras. In [4], the concept of derivation for lattices was introduced and some of its properties are investigated. For more details, the reader is referred to [3, 5–7].

Iséki and Tanaka introduced two classes of abstract algebras: BCK-algebras and BCI-algebras [8, 9]. Neggers and Kim introduced the notion of *d*-algebras which is another useful generalization of BCK-algebras and then investigated several relations between *d*-algebras and BCK-algebras as well as several other relations between *d*-algebras and oriented digraphs [10]. Kim and Neggers [11] introduced the notion of Bin(*X*) and obtained a semigroup structure. Bell and Kappe [1] studied rings in which derivations satisfy certain algebraic conditions. Alshehri [12] applied the notion of derivations in incline algebras.

The present authors [13] introduced the notion of ranked bigroupoids and discussed (*X*, ∗, *ω*)-self-(co)derivations. In addition, they defined rankomorphisms and (*X*, ∗, *ω*)-scalars for ranked bigroupoids and obtained some properties of these as well. Recently, Jun et al. [14] obtained further results on derivations of ranked bigroupoids, and Jun et al. [15] introduced the notion of generalized coderivations in ranked bigroupoids and showed that new generalized coderivations of ranked bigroupoids are obtained by combining a generalized self-coderivation with a rankomorphism.

In this paper, we extend the theory of derivations on a ranked bigroupoid to that of a type of derivation on ranked trigroupoids, that is, two-step derivations on ranked trigroupoids (*X*, ∗, •, ◊) considered as a couple of ranked bigroupoids (*X*, ∗, •) and (*X*, •, ◊) with *d* : *X* → *X* such a two-step derivation on (*X*, ∗, •, ◊) if it is a self-derivation on both (*X*, ∗, •) and (*X*, •, ◊). The role of the operation • in this definition is the more interesting one since it acts as the minor operation in (*X*, ∗, •) and the major operation in (*X*, •, ◊). From the results obtained below it is clear that it is indeed possible to obtain meaningful insights, especially via the notion of a couplet (*D*, *d*) on a ranked trigroupoid consisting of a pair of mappings *D*, *d* : *X* → *X* satisfying a natural condition (6) stated below which arises in a rather natural way from the context and is seen to be of interest in this study and presumably of any followup as well.

## 2. Preliminaries

An *d-algebra* [10] is a nonempty set *X* with a constant 0 and a binary operation “∗” satisfying the following axioms:

-  
*x*∗*x* = 0,
- 0∗*x* = 0,
-  
*x*∗*y* = 0 and *y*∗*x* = 0 imply *x* = *y* for all *x*, *y* ∈ *X*.

A BCK-algebra is a *d*-algebra *X* satisfying the following additional axioms:

(D) ((*x*∗*y*)∗(*x*∗*z*))∗(*z*∗*y*) = 0,
(E) (*x*∗(*x*∗*y*))∗*y* = 0 for all *x*, *y*, *z* ∈ *X*.

Given a nonempty set *X*, we let Bin(*X*) denote the collection of all groupoids (*X*, ∗), where ∗ : *X* × *X* → *X* is a map and where ∗(*x*, *y*) is written in the usual product form. Given elements (*X*, ∗) and (*X*, •) of Bin(*X*), define a product “□” on these groupoids as follows:

(1) $$\begin{array}{c} \;\left(X,*\right)□\left(X,•\right)=\left(X,□\right), \end{array}$$

where

(2) $$\begin{array}{c} x\;□\;y=\left(x*y\right)•\left(y*x\right) \end{array}$$

for any *x*, *y* ∈ *X*. Using that notion, Kim and Neggers proved the following theorem.

**Theorem 1 (see [11]).**

( Bin (*X*), □) is a semigroup; that is, the operation “□” as defined in general is associative. Furthermore, the left-zero-semigroup is the identity for this operation.

A* ranked bigroupoid* is an algebraic system (*X*, ∗, •) where *X* is a nonempty set and “∗” and “•” are binary operations defined on *X*. We may consider the first binary operation ∗ as the* major operation,* and the second binary operation • as the* minor operation*.

**Example 2 (see [16]).**

A *K*-algebra is defined as an algebraic system (*G*, •, ⊙) where (*G*, •) is a group and where *x*⊙*y* : = *x*•*y* ^−1^. Hence every *K*-algebra is a ranked bigroupoid.

**Example 3 (see [13]).**

We construct a ranked bigroupoid from any BCK-algebra. In fact, given a BCK-algebra (*X*, ∗, 0), if we define a binary operation “∧” on *X* by *x*∧*y* : = *x*∗(*x*∗*y*) for any *x*, *y* ∈ *X*, then (*X*, ∗, ∧) is a ranked bigroupoid.

We introduce the notion of “ranked bigroupoids” to distinguish two bigroupoids (*X*, ∗, •) and (*X*, •, ∗). Even though (*X*, ∗, •) = (*X*, •, ∗) in the sense of bigroupoids, the same is not true in the sense of ranked bigroupoids. This is analogous to the situation for sets, where {*x*, *y*} = {*y*, *x*} but 〈*x*, *y*〉 ≠ 〈*y*, *x*〉 in general.

Given an element (*X*, ∗) ∈ Bin(*X*), (*X*, ∗) has a natural associated ranked bigroupoid (*X*, ∗, ∗); that is, the major operation and the minor operation coincide.

Given a ranked bigroupoid (*X*, ∗, *ω*), a map *d* : *X* → *X* is called an (*X*, ∗, *ω*)-*self-derivation* if for all *x*, *y* ∈ *X*,

(3) $$\begin{array}{c} d\left(x*y\right)=\left(d\left(x\right)*y\right)\omega \left(x*d\left(y\right)\right). \end{array}$$

In the same setting, a map *d* : *X* → *X* is called an (*X*, ∗, *ω*)-*self-coderivation* if for all *x*, *y* ∈ *X*,

(4) $$\begin{array}{c} d\left(x*y\right)=\left(x*d\left(y\right)\right)\omega \left(d\left(x\right)*y\right). \end{array}$$

Note that if (*X*, *ω*) is a commutative groupoid, then (*X*, ∗, *ω*)-self-derivations are (*X*, ∗, *ω*)-self-coderivations. A map *d* : *X* → *X* is called an* abelian-*(*X*, ∗, *ω*)*-self-derivation* if it is both an (*X*, ∗, *ω*)-self-derivation and an (*X*, ∗, *ω*)-self-coderivation.

## 3. Two-Step Derivations and Couplets on Trigroupoids

An algebraic system (*X*, ∗, •, ◊) is said to be a* ranked trigroupoid* if algebraic systems (*X*, ∗, •) and (*X*, •, ◊) are ranked bigroupoids. A* two-step derivation* on a ranked trigroupoid (*X*, ∗, •, ◊) is a mapping *d* : *X* → *X* such that *d* is both an (*X*, ∗, •)-self-derivation and an (*X*, •, ◊)-self-derivation.

Obviously, if one considers ranked *n*-groupoids (*X*, ∗_1_, ∗_2_,…, ∗_*n*_), then one may consider (*n* − 1)-step derivations *d* : *X* → *X* for which one has *d* as an (*X*, ∗_*k*_, ∗_*k*+1_)-self-derivation for *k* = 1,…, *n* − 1.

In this paper we will mostly be interested in the case of two-step-derivations on ranked trigroupoids and some related pairs of maps (*D*, *d*) which we call* couplets*. For ranked *n*-groupoids where *n* ≥ 4, we obtain triplets, quadruplets, and so forth, as the appropriate generalizations.

Let *d* : *X* → *X* be a two-step derivation on a ranked trigroupoid (*X*, ∗, •, ◊). Then, for any *x*, *y* ∈ *X*, we have

(5) d2(x∗y) =d(d(x∗y)) =d[(d(x)∗y)•(x∗d(y))] =[d(d(x)∗y)•(x∗d(y))]  ◊[(d(x)∗y)•d(x∗d(y))] =[{(d2(x)∗y)•(d(x)∗d(y))}•(x∗d(y))]  ◊[(d(x)∗y)•[(d(x)∗d(y))•(x∗d2(y))]].

If we let *D* : = *d* ^2^, then it follows that

(6) D(x∗y) =[{(D(x)∗y)•(d(x)∗d(y))}•(x∗d(y))] ◊[(d(x)∗y)•[(d(x)∗d(y))•(x∗D(y))]].

We call (*D*, *d*) a* couplet* on a ranked trigroupoid (*X*, ∗, •, ◊) if it satisfies condition (6), and *D*(0) = 0 if *X* contains a constant 0.

**Example 4 .**

Let **R** be the set of all real numbers and let (**R**, ·, +, −) be the ranked trigroupoid where ·, +, and  − are usual multiplication, addition, and subtraction, respectively. If we let (*D*, *d*) be a couplet on the ranked trigroupoid (**R**, ·, +, −), then

(7) D(x·y)=[D(x)y+d(x)d(y)+xdy] −[d(x)y+d(x)d(y)+xD(y)]=[D(x)y−xD(y)]+[xd(y)−d(x)y].

If we let *y* : = *x* in (7), then

(8) $$\begin{array}{c} D\left(x^{2}\right)=\left[D\left(x\right)x-xD\left(x\right)\right]+\left[xd\left(x\right)-d\left(x\right)x\right]=0. \end{array}$$

Thus *D*(*x* ^2^) = 0 for all *x* ∈ **R**, whence *D*(*t*) = 0 for all *t* ≥ 0. If we let *y* : = 1 in (7), then *D*(*x*) = [*D*(*x*) − *xD*(1)]+[*xd*(1) − *d*(*x*)] = *D*(*x*) + *xd*(1) − *d*(*x*). It follows that, for any *x* ∈ **R**,

(9) $$\begin{array}{c} d\left(x\right)=xd\left(1\right), \end{array}$$

which implies that *xd*(*y*) − *d*(*x*)*y* = *xyd*(1) − *xd*(1)*y* = 0. Hence, by (7), we have *D*(*xy*) = *D*(*x*)*y* − *xD*(*y*) + *xd*(*y*) − *d*(*x*)*y* = *D*(*x*)*y* − *xD*(*y*); that is,

(10) $$\begin{array}{c} D\left(x\cdot y\right)=D\left(x\right)y-xD\left(y\right). \end{array}$$

If we let *x* < 0 and *y* : = −1 in (9), then we have 0 = *D*(−*x*) = *D*(*x*)·(−1) − *xD*(−1). Hence *D*(*x*) = −*xD*(−1) for all *x* < 0.

In Example 4, if *d* is a two-step derivation on (**R**, ·, +, −), then *d*(*xy*) = *d*(*x*)*y* + *xd*(*y*) and *d*(*x* + *y*) = (*d*(*x*) + *y*)−(*x* + *d*(*y*)) for any *x*, *y* ∈ **R**. It follows that *d*(2*x*) = *d*(*x* + *x*) = (*d*(*x*) + *x*)−(*x* + *d*(*x*)) = 0, which proves that *d*(*x*) = *d*(2 · (*x*/2)) = 0 for all *x* ∈ **R**.

**Example 5 .**

Let *X* : = [0, *∞*) and let (*X*, ·, +, +) be a ranked trigroupoid where ·, + are usual multiplication and addition, respectively. Define a map *φ* : *X* → *X* satisfying *φ*(*a* + *b*) = *φ*(*a*) + *φ*(*b*) for all *a*, *b* ∈ *X*. For *k* > 0, *k* ≠ 1, *λ* ∈ **R**, we define a map *D* : *X* → *X* by *D*(*x*): = *φ*(*a*)*k* ^*a*+*λ*^ where *x* = *k* ^*a*^ and *D*(0) = 0. Then *D* is a (*X*, ·, +)-self-derivation. In fact, if *x* = *k* ^*a*^, *y* = *k* ^*b*^ for some *a*, *b* ∈ *X*, then

(11) D(x·y)=D(ka·kb)=φ(a+b)ka+b+λ=[φ(a)+φ(b)]ka+b+λ=[φ(a)ka+λ]kb+[φ(b)kb+λ]ka=D(x)y+xD(y).

Assume that (*D*, *d*) is a couplet on the ranked trigroupoid (*X*, ·, +, +) for some self-(*X*, +, +)-derivation *d*. Then,

(12) D(x·y)=D(x)y+d(x)d(y)+xd(y)+d(x)y +d(x)d(y)+xD(y).

Since *D*(*x* · *y*) = *D*(*x*)*y* + *xD*(*y*), we obtain

(13) $$\begin{array}{c} 0=d\left(x\right)\left[y+d\left(y\right)\right]+\left[x+d\left(x\right)\right]d\left(y\right). \end{array}$$

**Proposition 6 .**

There is no two-step derivation on the ranked trigroupoid (**R**, ·, −, +).

**Proof**

Assume that *d* : **R** → **R** is a two-step derivation on (**R**, ·, −, +). Then, for any *x*, *y* ∈ **R**,

(14) $$\begin{array}{c} d\left(x\cdot y\right)=d\left(x\right)y-xd\left(y\right), \end{array}$$

(15) $$\begin{array}{c} d\left(x-y\right)=\left(d\left(x\right)-y\right)+\left(x-d\left(y\right)\right). \end{array}$$

If we let *y*∶ = −*x* in (15), then

(16) d(2x)=(d(x)−(−x))+(x−d(−x))=2x+(d(x)−d(−x)),

(17) d(−x2)=d(x)·(−x)−x·d(−x)=−x·d(x)−x·d(−x).

If we let *x* : = 0 in (16), then *d*(0) = *d*(2 · 0) = 2 · 0 + (*d*(0) − *d*(−0)) = 0. On the other hand, if we let *x* : = 1 and *y* : = 0 in (15), then *d*(1 − 0) = (*d*(1) − 0)+(1 − *d*(0)), proving that *d*(0) = 1 is a contradiction. Hence there is no two-step derivation on the ranked trigroupoid (**R**, ·, −, +).

**Proposition 7 .**

There is no two-step derivation on the ranked trigroupoid (**R**, ·, +, +).

**Proof**

Assume that *d* : **R** → **R** is a two-step derivation on (**R**, ·, +, +). Then, for any *x*, *y* ∈ **R**,

(18) $$\begin{array}{c} d\left(x\cdot y\right)=d\left(x\right)y+xd\left(y\right), \end{array}$$

(19) $$\begin{array}{c} d\left(x+y\right)=\left(d\left(x\right)+y\right)+\left(x+d\left(y\right)\right). \end{array}$$

If we let *y* : = 0 in (19), then *d*(*x* + 0) = *d*(*x*) + 0 + *x* + *d*(0) and hence *d*(0) = −*x* for all *x* ∈ **R**. If we let *y* : = 0, *x* : = 0 in (18), then −*x* = *d*(0 · 0) = *d*(0) · 0 + 0 · *d*(0) = 0, for any *x* ∈ **R**, is a contradiction.

**Proposition 8 .**

Let (*D*, *d*) be a couplet on a ranked trigroupoid (**R**, ·, −, +). If *d*(1) ≠ 1/2 and *λ* : = (*D*(1) − *d*(1))/(2*d*(1) − 1), then *D*(*xy*) = *D*(*x*)*y* + *xD*(*y*) − 2*λ* ^2^ *xy* for all *x*, *y* ∈ **R**. In particular, if *D*(1) = *d*(1), then *D* is a (**R**, ·, +)-self-derivation.

**Proof**

If (*D*, *d*) is a couplet on a ranked trigroupoid (**R**, ·, −, +), then, for any *x*, *y* ∈ **R**, we have

(20) D(xy)=(D(x)y−d(x)d(y))−xd(y)+d(x)y −(d(x)d(y)−xD(y))=D(x)y+xD(y)−2d(x)d(y) +(d(x)y−xd(y)).

Since *d*(1) ≠ 1/2, if we let *y* : = 1 in (20), then *D*(*x*) = *D*(*x*) + *xD*(1) − 2*d*(*x*)*d*(1) + *d*(*x*) − *xd*(1). It follows that

(21) 0=xD(1)−2d(x)d(1)+d(x)−xd(1)=x[D(1)−d(1)]−[2d(1)−1]d(x).

Hence *d*(*x*) = (*D*(1) − *d*(1))/(2*d*(1) − 1)*x* = *λx* for any  *x* ∈ **R**. If we change *d*(*x*) into *λx* for any *x* ∈ **R**, then we obtain

(22) D(xy)=D(x)y+xD(y)−2(λx)(λy)+(λx)y−x(λy)=D(x)y+xD(y)−2λ2xy

for all *x*, *y* ∈ **R**. In particular, if *D*(1) = *d*(1), then *λ* = 0 and hence *D*(*xy*) = *D*(*x*)*y* + *xD*(*y*); that is, *D* is a (**R**, ·, +)-self-derivation.

## 4. Frame Algebras and *fr*(3)-Algebras

A groupoid (*X*, ∗, 0) is said to be a* frame algebra* if it satisfies the axioms (*A*), (*B*), and

(F)  
*x*∗0 = *x*,

for all *x* ∈ *X*.

**Example 9 .**

(1) Every BCK-algebra is a frame algebra.

(2) Every lattice implication algebra (see [17, 18]) is a frame algebra.

The collection of frame algebras includes the collection of BCK-algebras and it is a variety. In a frame algebra the element 0 is unique. Indeed, if 0_1_ and 0_2_ are both zeros, then *x* ≠ 0_1_, 0_2_ yields 0_1_ = *x*∗*x* = 0_2_.

**Proposition 10 .**

The collection of all frame algebras (*X*, ∗_*i*_, 0) forms a subsemigroup of the semigroup (Bin(*X*), □).

**Proof**

Given frame algebras (*X*, ∗, 0), (*X*, •, 0), if we let (*X*, □): = (*X*, ∗)  □  (*X*, •), then *x*  □  *y* = (*x*∗*y*)•(*y*∗*x*) for all *x*, *y* ∈ *X*. It follows that *x*  □  *x* = (*x*∗*x*)•(*x*∗*x*) = 0•0 = 0, 0  *x* = (0∗*x*)•(*x*∗0) = 0  •  *x* = 0, and *x*  □  0 = (*x*∗0)•(0∗*x*) = *x*•0 = *x*, proving that (*X*, □) is a frame algebra. This proves the proposition.

Given groupoids (*X*, ∗), (*X*, •) ∈ Bin(*X*), we define

(23) (X,∗)S(X,•)≔{(X,∇) ∣ ∀x,y∈X, x  ∇  y∈{x∗y,x•y}}.

**Proposition 11 .**

Let (*X*, ∗, 0) and (*X*, •, 0) be frame algebras. If (*X*, ∇)∈(*X*, ∗)*S*(*X*, •), then (*X*, ∇) is a frame algebra.

**Proof**

If (*X*, ∇)∈(*X*, ∗)*S*(*X*, •), then *x*  ∇  *y* ∈ {*x*∗*y*, *x*•*y*} for all *x*, *y* ∈ *X*. It follows that *x*  ∇  *x* ∈ {*x*∗*x*, *x*•*x*} = {0} implies that *x*  ∇  *x* = 0. Moreover, 0  ∇  *x* ∈ {0∗*x*, 0•*x*} = {0} shows that 0  ∇  *x* = 0, and *x*  ∇  0 ∈ {*x*∗0, *x*•0} = {*x*} shows that *x*  ∇  0 = *x*, proving that (*X*, ∇) is a frame algebra.

A ranked trigroupoid (*X*, ∗, •, ◊) is called an* fr*(3)*-algebra* if

(G) (*X*, ∗, 0_1_), (*X*, •, 0_2_), (*X*, ◊, 0_3_) are frame algebras,
(H) 0_1_ = 0_2_ = 0_3_.

**Theorem 12 .**

Let (*X*, ∗, •, ◊) be an* fr*(3)-algebra. If *d* : *X* → *X* is a two-step derivation on *X*, then

- *d*(0) = 0,
- *d*(*x*) = *d*(*x*)•*x* = *d*(*x*)  ◊  *x* for all *x* ∈ *X*.

**Proof**

(i) If *d* is a two-step derivation on (*X*, ∗, •, ◊), then for any *x*, *y* ∈ *x*, we have

(24) $$\begin{array}{c} d\left(x*y\right)=\left(d\left(x\right)*y\right)•\left(x*d\left(y\right)\right), \end{array}$$

(25) $$\begin{array}{c} d\left(x•y\right)=\left(d\left(x\right)•y\right)◊\left(x•d\left(y\right)\right). \end{array}$$

It follows that *d*(0) = *d*(0∗*y*) = (*d*(0)∗*y*)•(0∗*d*(*y*)) = (*d*(0)∗*y*)•0 = *d*(0)∗*y*; that is, *d*(0) = *d*(0)∗*y*, for all *y* ∈ *X*. If we let *y* : = *d*(0), then by applying (A) we obtain *d*(0) = *d*(0)∗*d*(0) = 0.

(ii) Given *x* ∈ *X*, we have *d*(*x*) = *d*(*x*∗  0) = (*d*(*x*)∗0)  •  (*x*∗*d*(0)) = *d*(*x*)•*x* and *d*(*x*) = *d*(*x*•  0) = (*d*(*x*)•0)  ◊  (*x*•*d*(0)) = *d*(*x*)  ◊  *x*.

Given a two-step derivation *d* on a trigroupoid (*X*, ∗, •, ◊), we denote its kernel by Ker⁡(*d*): = {*x* ∈ *X*∣*d*(*x*) = 0}.

**Proposition 13 .**

Let (*X*, ∗, •, ◊) be an fr(3)-algebra. If *d* : *X* → *X* is a two-step derivation on *X*, then

- *x*∗*d*(*x*), *x*•*d*(*x*) ∈ *Ker*(*d*),
- *x* ∈ *Ker*(*d*) implies that *x*∗*y*, *x*•*y* ∈ *Ker*(*d*),
- *Ker*(*d*)⊆*Ker*(*d* ^2^),

for all *x*, *y* ∈ *X*.

**Proof**

(i) If we let *y* : = *d*(*x*) in (24) and (25), respectively, then *d*(*x*∗*d*(*x*)) = (*d*(*x*)∗*d*(*x*))  •  (*x*∗*d*(*d*(*x*))) = 0  •  (*x*∗*d* ^2^(*x*)) = 0 an *d*(*x*•  *d*(*x*)) = (*d*(*x*)  •  *d*(*x*))  ◊  (*x*•  *d*(*d*(*x*))) = 0  ◊  (*x*•  *d* ^2^(*x*)) = 0, proving that *x*∗*d*(*x*), *x*•*d*(*x*) ∈ Ker⁡(*d*) for any *x* ∈ *X*.

(ii) If *x* ∈ Ker⁡(*d*), then *d*(*x*∗*y*) = (*d*(*x*)∗*y*)•(*x*∗*d*(*y*)) = (0∗*y*)•(*x*∗*d*(*y*)) = 0 and *d*(*x*•*y*) = (*d*(*x*)•*y*)  ◊  (*x*•  *d*(*y*)) = 0 for any *y* ∈ *X*, proving that *x*∗*y*,   *x*•*y* ∈ Ker⁡(*d*).

(iii) If *x* ∈ Ker⁡(*d*), then *d* ^2^(*x*) = *d*(*d*(*x*)) = *d*(0) = 0 by Theorem 12, which shows that *x* ∈ Ker⁡(*d* ^2^).

**Proposition 14 .**

Let (*X*, ∗, •, ◊) be an fr(3)-algebra. If *d* : *X* → *X* is a two-step derivation on *X*, then

(26) $$\begin{array}{c} x\in Ker\left(d^{2}\right)\;implies\;x*y\in Ker\left(d^{2}\right) \end{array}$$

for all *y* ∈ *X*.

**Proof**

If *x* ∈ Ker⁡(*d* ^2^), then *d*(*d*(*x*)) = 0 and hence

(27) d2(x∗y) =d(d(x∗y))=d[(d(x)∗y)•(x∗d(y))] =[d(d(x)∗y)•(x∗d(y))]  ◊[(d(x)∗y)•d(x∗d(y))] =[{(d(d(x)∗y))•(d(x)∗d(y))}•(x∗d(y))]  ◊[(d(x)∗y)•d(x∗d(y))] =0  ◊  [(d(x)∗y)•d(x∗d(y))]=0,

for all *y* ∈ *X*, which proves that *x*∗*y* ∈ Ker⁡(*d* ^2^).

Note that *d* ^2^(*x*•*y*) may not be computable unless the behavior of *d*(*u*  ◊  *v*) is specified, since *d* ^2^(*x*•*y*) = *d*((*d*(*x*)•*y*)  ◊  (*x*•*d*(*y*))) = *d*(*u*  ◊  *v*) for some *u*, *v* ∈ *X*.

**Proposition 15 .**

Let (*X*, ∗, •, ◊) be an fr(3)-algebra. If (*D*, *d*) is a couplet of (*X*, ∗, •, ◊), then *Ker*(*D*)∗*X*⊆*Ker*(*D*).

**Proof**

If (*D*, *d*) is a couplet of (*X*, ∗, •, ◊) and if *x* ∈ Ker⁡(*D*), then *D*(*x*)∗*y* = 0∗*y* = 0 for any *y* ∈ *X*. It follows from (6) that *D*(*x*∗*y*) = 0  ◊  [(*d*(*x*)∗*y*)•[(*d*(*x*)∗*d*(*y*))•(*x*∗*D*(*y*))]] = 0, proving that *x*∗*y* ∈ Ker⁡(*D*).

Let (*X*, ≤) be a poset with minimal element 0. Define a binary operation “∗” on *X* by

(28) $$\begin{array}{c} x*y=\{\begin{array}{cc} 0, & \text{if}\;\;x\leq y,\\ x, & \text{otherwise}. \end{array} \end{array}$$

Then (*X*, ∗, 0) is a BCK-algebra, called a* standard BCK-algebra* inherited from the poset (*X*, ≤).

**Proposition 16 .**

Let (*X*, ∗, 0) be a standard BCK-algebra. Let (*X*, ∗, •, ◊) be an* fr*(3)-algebra and let (*D*, *d*) be a couplet of (*X*, ∗, •, ◊). If *D*(*x*∗*y*) ≠ 0 for some *x*, *y* ∈ *X*, then *D*(*x*∗*y*) = *D*(*x*)∗*y* = *D*(*x*) and *x*∗*y* = *x*.

**Proof**

Let *D*(*x*∗*y*) ≠ 0. We claim that *D*(*x*)∗*y* ≠ 0. Suppose that *D*(*x*)∗*y* = 0. Since (*X*, ∗, •, ◊) is a* fr*(3)-algebra, by applying (6), we obtain that *D*(*x*∗*y*) = 0 is a contradiction. Since (*X*, ∗, 0) is a standard BCK-algebra, we obtain *D*(*x*)∗*y* = *D*(*x*). We claim that *x*∗*y* = *x*. If *x*∗*y* = 0, then *D*(*x*∗*y*) = *D*(0) = 0 is a contradiction. It follows that *D*(*x*∗*y*) = *D*(*x*) = *D*(*x*)∗*y*, proving the proposition.

## 5. Classification of Linear Ranked Trigroupoids

Let *X* : = **R** be the real field and let (*X*, ∗, •, ◊) be a ranked trigroupoid, where (*X*, ∗), (*X*, •), and  (*X*, ◊) are linear groupoids; that is, *x*∗*y* : = *A* + *Bx* + *Cy*, *x*•*y* : = *a* + *bx* + *cy*, and *x*  ◊  *y* : = *α* + *βx* + *γy* for any *x*, *y* ∈ *X*, where *A*, *B*, *C*, *a*, *b*, *c*, *α*, *β*, *γ* ∈ *X* (fixed).

Thus, if *d* : *X* → *X* is a two-step derivation on (*X*, ∗, •, ◊), then for any *x*, *y* ∈ *X*, we have

(29) d(x∗y)=(d(x)∗d(y))•(x∗d(y))=(A+Bd(x)+Cy)•(A+Bx+Cd(y))=a+(b+c)A+B(cx+bd(x))+C(by+cd(y))

and also in a similar manner we obtain

(30) d(x•y)=α+(β+γ)a+b(γx+βd(x)) +c(βy+γd(y)).

If *d*(*x*) = 0 for all *x* ∈ *X*, then (29) and (30) become

(31) a+(b+c)A+Bcx+Cby=0,α+(β+γ)a+bγx+cβy=0

for any *x*, *y* ∈ *X*. If we let *x* : = 0, *y* : = 0 in (31), then we obtain *a* + (*b* + *c*)*A* = 0,  *α* + (*β* + *γ*)*a* = 0. Hence, (31) reduces to

(32) $$\begin{array}{c} Bcx+Cby=0, \end{array}$$

(33) $$\begin{array}{c} b\gamma x+c\beta y=0 \end{array}$$

for all *x*, *y* ∈ *X*. If we let *x* : = 1,  *y* : = 0 and let *x* : = 0,  *y* : = 1 in (32) and (33), respectively, then we obtain

(34) $$\begin{array}{c} Bc=0,\;\;Cb=0,\;\;b\gamma =0,\;\;c\beta =0. \end{array}$$

From this information, we obtain the following propositions.

**Proposition 17 .**

Let *X* : = **R** be the real field and let (*X*, ∗, •, ◊) be a ranked trigroupoid, where (*X*, ∗), (*X*, •), (*X*, ◊) are linear groupoids; that is, *x*∗*y* : = *A* + *Bx* + *Cy*, *x*•*y* : = *a* + *bx* + *cy*, and *x*  ◊  *y* : = *α* + *βx* + *γy* for any *x*, *y* ∈ *X*, where *A*, *B*, *C*, *a*, *b*, *c*, *α*, *β*, *γ* ∈ *X* (fixed). Let *d* : *X* → *X* be a two-step derivation such that *d*(*x*) = 0 for all *x* ∈ *X*.

1. If *bc* ≠ 0 and *b* + *c* ≠ 0, then *x*∗*y* = −*a*/(*b* + *c*), *x*•*y* : = *a* + *bx* + *cy*, *x*  ◊  *y* = 0;
2. If *bc* ≠ 0 and *b* + *c* = 0, then *x*∗*y* = *A*, *x*•*y* = *b*(*x* − *y*), *x*  ◊  *y* = 0.

**Proof**

(i) If *bc* ≠ 0, then it follows from (34) that we obtain *B* = *C* = 0, *β* = *γ* = *α* = 0. If *b* + *c* ≠ 0, then we have *x*∗*y* = −*a*/(*b* + *c*) and *x*  ◊  *y* = 0.

(ii) If *bc* ≠ 0 and *b* + *c* = 0, then 0 = *a* + (*b* + *c*)*A* = *a* and *A* is arbitrary, and hence we obtain the result.

**Proposition 18 .**

Let *X* : = **R** be the real field and let (*X*, ∗, •, ◊) be a ranked trigroupoid, where (*X*, ∗), (*X*, •), (*X*, ◊) are linear groupoids; that is, *x*∗*y* : = *A* + *Bx* + *Cy*, *x*•*y* : = *a* + *bx* + *cy*, and *x*   ◊  *y* : = *α* + *βx* + *γy* for any *x*, *y* ∈ *X*, where *A*, *B*, *C*, *a*, *b*, *c*, *α*, *β*, *γ* ∈ *X* (fixed). Let *d* : *X* → *X* be a two-step derivation such that *d*(*x*) = 0 for all *x* ∈ *X*:

- if *b* = *c* = 0 and *b* + *c* ≠ 0, then *x*∗*y* = *A* + *Bx* + *Cy*, *x*•*y* : = 0, *x*  ◊  *y* = *βx* + *γy*;
- if *b* ≠ 0, *c* = *β* = 0, then *x*∗*y* = −*a*/*b* + *Bx*, *x*•*y* = *a* + *bx*, *x*  ◊  *y* = 0;
- if *b* ≠ 0, *c* = 0, *β* ≠ 0, then *x*∗*y* = *α*/*bβ* + *Bx*, *x*•*y* = −*α*/*β* + *bx*, *x*  ◊  *y* = *α* + *βy*;
- if *b* = 0, *c* ≠ 0, *γ* = 0, then *x*∗*y* = −*a*/*c* + *Cy*, *x*•*y* = *a* + *cy*, *x*  ◊  *y* = 0;
- if *b* = 0, *c* ≠ 0, *γ* ≠ 0, then *x*∗*y* = *α*/*cγ* + *Cy*, *x*•*y* = −*α*/*γ* + *cy*, *x*  ◊  *y* = *α* + *γy*.

**Proof**

The proof is similar to Proposition 17 and we omit it.

In Propositions 17 and 18, we observe that there are 6 different types of linear ranked trigroupoids in the special case of *d*(*x*) = 0 for all *x* ∈ *X*, and most of them are classified by the properties of *b*, *c* in *x*•*y* = *a* + *bx* + *cy*.

## 6. Conclusion

The notion of two-step derivations is a generalization of derivations. This leads to the study of trigroupoids, and we explore some relations with several algebras, for example, BCK-algebras, frame algebras, and so forth. The classification of linear ranked trigroupoids then explains a number of concrete algebraic structures with derivations.

## References

[1] Bell HE, Kappe L-C (1989). Rings in which derivations satisfy certain algebraic conditions. *Acta Mathematica Hungarica*.

[2] Posner EC (1957). Derivations in prime rings. *Proceedings of the American Mathematical Society*.

[3] Jun YB, Xin XL (2004). On derivations of BCI-algebras. *Information Sciences*.

[4] Szász G (1975). Derivations of lattices. *Acta Universitatis Szegediensis. Acta Scientiarum Mathematicarum*.

[5] Çeven Y (2009). Symmetric bi-derivations of lattices. *Quaestiones Mathematicae. Journal of the South African Mathematical Society*.

[6] Kaya K (1988). Prime rings with-derivations. *Hacettepe Bulletin of Natural Sciences and Engineering*.

[7] Zhan J, Liu YL (2005). On *f*-derivations of BCI-algebras. *International Journal of Mathematics and Mathematical Sciences*.

[8] Iséki K (1980). On BCI-algebras. *Mathematics Seminar Notes*.

[9] Iséki K, Tanaka S (1978/79). An introduction to the theory of BCK-algebras. *Mathematica Japonica*.

[10] Neggers J, Kim HS (1999). On *d*-algebras. *Mathematica Slovaca*.

[11] Kim HS, Neggers J (2008). The semigroups of binary systems and some perspectives. *Bulletin of the Korean Mathematical Society*.

[12] Alshehri NO (2010). On derivations of incline algebras. *Scientiae Mathematicae Japonicae*.

[13] Alshehri NO, Kim HS, Neggers J (2013). Derivations on ranked bigroupoids. *Applied Mathematics & Information Sciences*.

[14] Jun YB, Kim HS, Roh EH (2012). Further results on derivations of ranked bigroupoids. *Journal of Applied Mathematics*.

[15] Jun YB, Lee KJ, Park CH (2012). Coderivations of ranked bigroupoids. *Journal of Applied Mathematics*.

[16] Dar KH, Akram M (2004). Characterization of a *K*(*G*)-algebra by self maps. *Southeast Asian Bulletin of Mathematics*.

[17] Jun YB (1997). Implicative filters of lattice implication algebras. *Bulletin of the Korean Mathematical Society*.

[18] Xu Y (1993). Lattice implication algebras. *Journal of SouthWest JiaoTong University*.

